# Changing phase relationship of the stepping rhythm to neuronal oscillatory theta activity in the septo-hippocampal network of mice

**DOI:** 10.1007/s00429-020-02031-8

**Published:** 2020-02-14

**Authors:** Abhilasha Joshi, Peter Somogyi

**Affiliations:** 1 Department of Pharmacology, University of Oxford, Mansfield Road, Oxford, OX1 3QT UK; 2 Present Address: Department of Physiology, Kavli Institute for Fundamental Neuroscience, University of California, San Francisco, USA

**Keywords:** Movement, Medial septum, Stepping rhythm, Theta oscillations, Hippocampus, Navigation

## Abstract

Movement-related sensory and motor activity in the brain contributes to cognitive processes. We have observed that the frequency of stepping rhythm in head-fixed mice running on a jetball overlaps with the range of frequencies that characterize hippocampal rhythmic slow activity, including theta (~ 3 to 10 Hz). On average, step-cycle troughs (i.e. when the paw touches the ground) were weakly coupled to hippocampal theta oscillations. This weak coupling was sustained during a range of running speeds. In short temporal windows, step-cycle troughs were synchronous with hippocampal theta oscillatory cycle troughs, while during other periods they led or lagged behind theta cycles. Furthermore, simultaneously recorded theta rhythmic medial septal neurons in the basal forebrain were phase-coupled to both step-cycles and theta-cycles. We propose that the weak overall phase relationship of step-cycles with theta-cycles signifies a distinct mode of information processing. Transient synchronization of the step-cycle with theta may indicate the engagement of septo-hippocampal-entorhinal network with the current heading of the animal.

**Electronic supplementary material:**

The online version of this article (10.1007/s00429-020-02031-8) contains supplementary material, which is available to authorized users.

## Introduction

Interaction of animals with the environment involves movements, such as locomotion, sniffing, respiration, licking, grooming, rearing and chewing. Neurons across the entire temporal cortex, including those in the hippocampus, are responsive to locomotor variables, such as speed or direction of heading in both rodents (Alcantara et al. 1998; Czurkó et al. 2011; Kropff et al. 2015; McNaughton et al. 1983) and humans (Jacobs et al. 2007). Recent studies suggest that in addition to locomotion, other bodily movements are also represented by neuronal activity throughout cortical and subcortical areas (Musall et al. 2019; Tort et al. 2018). Translocation of the body through steps is a primary means by which an animal explores the world and executes behavior and stepping is a well-characterized rhythmic activity generated in the central nervous system (Grillner and Wallen 1985). Movement influences perception and cognitive processes (Gothe et al. 2018; Phillips-Silver and Trainor 2005), and they interact through multiple neuronal mechanisms. Indeed, the interaction of rhythmic muscle activity during breathing or dancing has been long recognized to influence cognitive processes (Gothe et al. 2018; Dahl et al. 2015). To explore rhythmic neuronal activity in the interconnected septo-temporal cortex system, we explored relationships between individual step-cycles and neuronal activity in the septum and hippocampus. Our observations suggest that there is a variable inter-relationship between movement parameters and neuronal activity in the septo-hippocampal system, which may explain the influence of self-motion on hippocampus-dependent behavior (Fattahi et al. 2018).

## Results

**Fig. 1:**
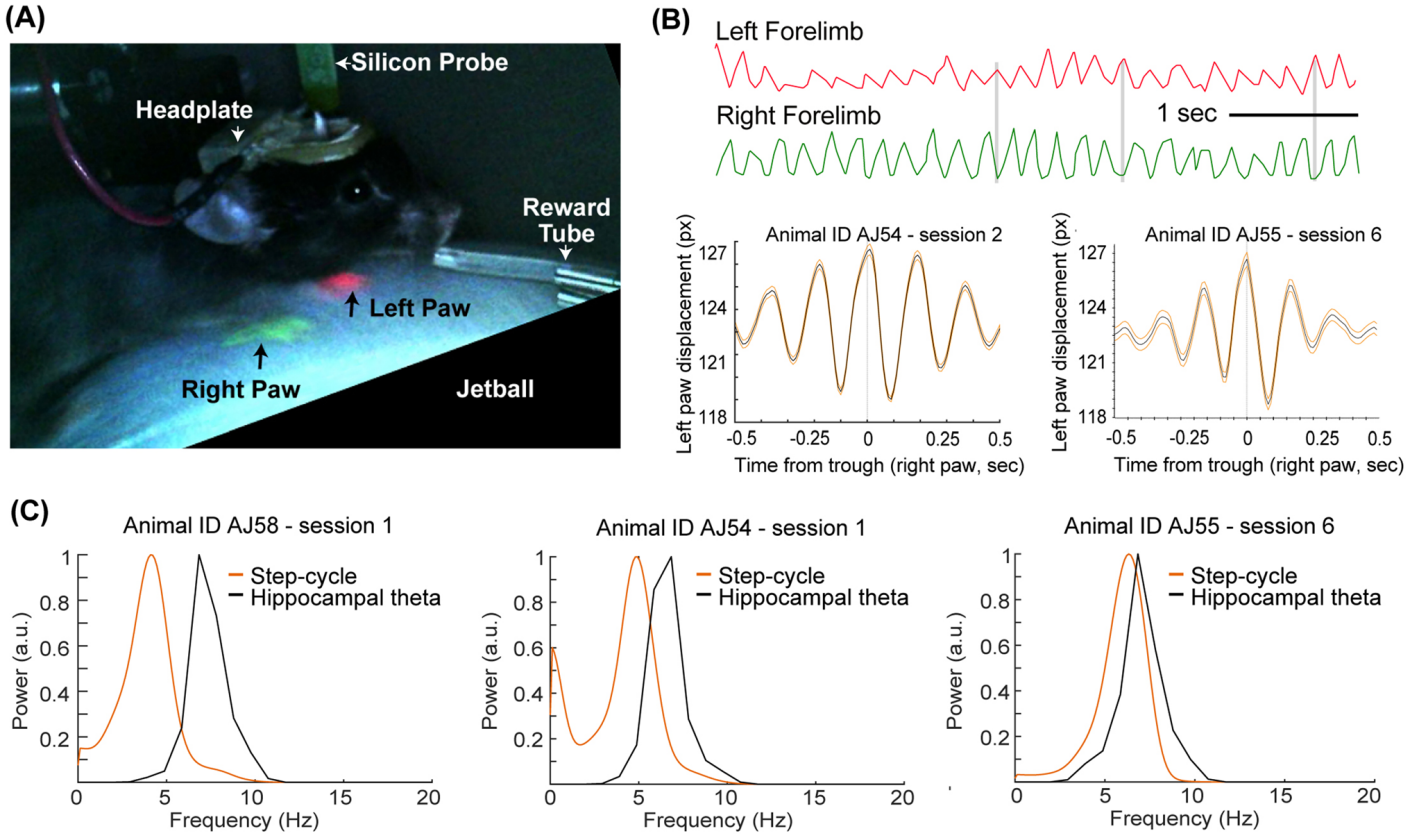
The frequency of stepping rhythm overlaps with hippocampal theta oscillations. **a** The forelimbs of head-fixed mice are tracked to monitor the stepping rhythm during running in a linear maze in virtual reality. Paws are marked with UV-glow body paint (green and red) and the color signal is tracked using a Pixy-camera. **b** This color-based monitoring method reliably captures alternating gait-cycles of the left and right forelimb. **c** Normalized power spectra of single paw step-cycles and hippocampal theta activity shows the variable overlap in the frequency of stepping and theta rhythm

Stepping rhythm of head-fixed mice (*n* = 3) navigating in a virtual linear maze for a sucrose reward was recorded by monitoring the displacement of front paws marked with body paint (Fig. 1a) using a Pixy camera (Nashaat et al. 2017). This method reliably captures the alternating left–right limb motion (Fig. 1b). During these recordings, hippocampal theta oscillatory activity, medial septal local field potential and neuronal units were recorded simultaneously to analyze their interrelationships with the stepping rhythm. Power spectral analysis revealed that the stepping rhythm ranged from ~ 3 to ~ 8 Hz during running (Fig. 1c), overlapping with rodent hippocampal and medial septal theta oscillatory activity (Nerad and McNaughton 2006). The number of steps (each paw taken separately) correlated with the running speed of the animal (as measured in arbitrary units of the moving virtual reality, median *r*, 0.5; Interquartile range 0.46–0.53, *n* = 3 animals, *p* < 0.001). Furthermore, the stepping rhythm of the right forepaw was monitored using another method, DeeplabCut (Mathis et al. 2018) to reveal the range of running cycle frequencies in head-fixed mice. Power spectral density analysis of three example recording sessions reveal the variable peak frequency of the stepping rhythm (Fig. 1c).

**Fig. 2:**
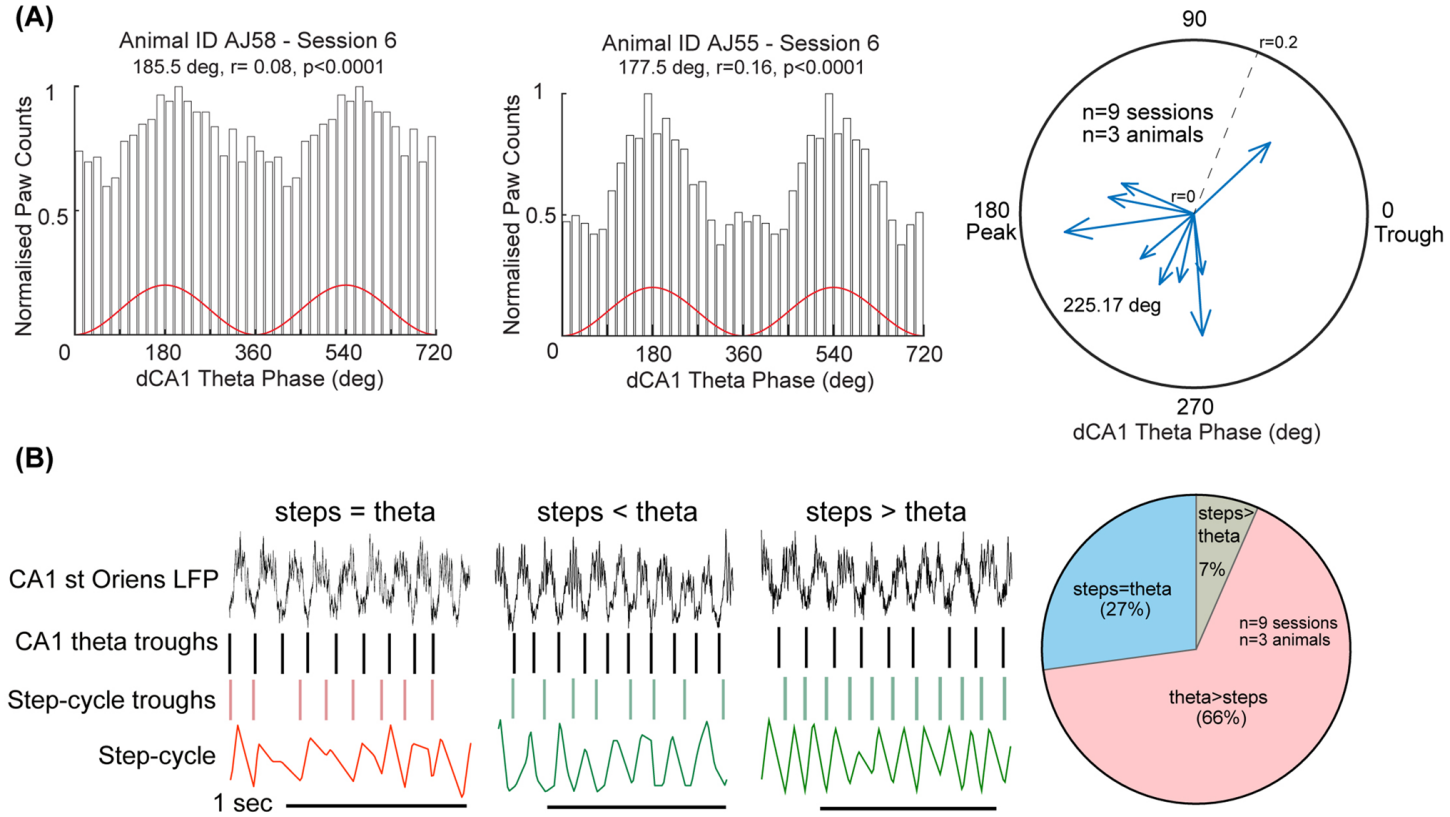
Relationship of step-cycle troughs to simultaneously recorded hippocampal theta oscillatory cycles. **a** (Left, middle) Phase relationship of step-cycle trough events, when the paw touches the ground (i.e. surface of the jetball), to hippocampal theta cycles during RUN periods. On average, there is a weak-correlation of the step-cycle troughs to hippocampal theta troughs. (Right) Preferential theta phase of step-cycle troughs with the depth of modulation (*r*) as radius during RUN periods for each recording session. Note, the preferential distribution of mean phases towards the peak/descending phase of theta. **b** (Left) Examples of epochs showing that the number of step-cycle troughs may be equal to, less than or more than the number of ongoing theta-cycle troughs. Black vertical lines, dorsal CA1 hippocampal theta troughs; red/green vertical lines, left/right forelimb troughs; colored traces, paw cycle captured by PixyMon. (Right) Percentage of 500 ms segments in which step-cycle troughs were equal to, fewer or more than the number of theta troughs. Values are averages for 9 sessions

Next, we studied if the step-cycles were related to ongoing hippocampal theta oscillatory cycles. Troughs of the step-cycles, i.e. when individual paws touch the ground, were plotted relative to the phase of theta cycles recorded in strata pyramidale or oriens in hippocampal CA1. On average, we observed a weak coupling of the two signals, as evidenced by mean vector lengths (group mean ± SD = 0.10 ± 0.03, *n* = 3 animals, three sessions per animal, *p* < 0.05, individual animal means; AJ54 = 0.10, AJ55 = 0.11, AJ58 = 0.09, Fig. 2a). Step-cycle troughs were used from the right forepaw for all the analyses. Interestingly, the step-cycle troughs corresponding to the right forepaw preferred the descending phase of theta (mean angular direction = 226°, *n* = 9 sessions) i.e. after the maximum input arriving from entorhinal cortex at the peak of theta and just before the maximum input from CA3 at the trough of theta (Mizuseki et al. 2009), though across multiple cycles they could occur at different phases. To test if the coupling of theta and paw cycles occurs by chance, we used a shuffling test (see "Methods"). For each recording session (*n* = 9), the value of the observed mean vector length was greater than the 99th percentile of shuffled vector length distributions. This suggests that the relationship between paw movement cycles and theta activity in the hippocampus is not due to a chance coupling between two oscillators.

To evaluate behavioral state-dependent variability in the coupling of step-cycle to hippocampal theta activity, we parsed the running data into three categories, VRon, VRoff and VRrwd (see "Methods"). We observed coupling of the step-cycle to theta in all periods (*n* = 4 recording sessions, Rayleigh test, *p* < 0.05), which met our criteria (i.e. excluding parsed data with < 200 step-cycle troughs). Furthermore, there was no detectable difference between these categories of running in the value of coupling, as estimated by comparing the mean vector lengths (*p* > 0.05, Kruskal–Wallis test). This suggests that, in our simple behavioral paradigm, there is a weak coupling of step-cycle troughs to cortical theta oscillatory activity that is not explained by a differential relationship during VRon, VRoff or VRrwd periods.

In rodents, theta frequency is weakly correlated with running speed (e.g. in rats it ranges from ~ 8.4 to ~ 9.2 Hz, Jeewajee et al. 2008). To evaluate if the step-theta relationship we observe varies differentially during slow and fast running speeds, we segmented the running data into three speed bins: slow (2–7 cm/s), medium (7–13 cm/s) and fast (13–19 cm/s). We observed a consistent coupling of step-cycle troughs and theta activity in the hippocampus in all three running periods (*n* = 5 recording sessions, Rayleigh test, *p* < 0.05). We did not find differences in the strength of theta coupling between different running speeds (*p* > 0.05, Kruskal–Wallis test). This suggests that the weak step-theta relationship is not due to the presence of a dominant running speed range and is prevalent through all the tested behaviors. Further studies that test the step-theta relationship in freely running animals may reveal differences depending on running speed.

In short temporal segments (Fig. 2b), step-cycle troughs could be equal to, less or higher than theta oscillatory cycle troughs. We evaluated if periods in which step-cycle troughs were equal to theta-cycle troughs had a different degree of coupling compared to periods with a different number of troughs. We divided the entire running data sequentially into 500 ms segments and calculated the proportion of segments in which the number of steps and theta troughs were equal, greater (theta trough > step trough) or less (theta trough < step trough). Overall, for all animals and sessions combined, in the majority of segments (65%) theta frequency was higher than step frequency. In a significant proportion of segments (27%), the number of theta troughs was equal to the number of paw cycle troughs. Paw cycle troughs were more frequent than theta troughs in only a small number of time segments (6% of intervals). This is consistent with our observation about the stepping rhythm frequency ranging from 3 to 8 Hz. We then compared the mean vector lengths between these three groups of temporal segments (step = theta, steps < theta, steps > theta). In each condition, we found that step-cycle troughs were coupled to theta activity (Rayleigh test), but we did not detect a difference between segment groups (*n* = 4 recording sessions, Kruskal–Wallis test *p* > 0.05). Overall, the above analyses suggest a weak but consistent coupling of step-cycle troughs to ongoing hippocampal theta activity.

**Fig. 3:**
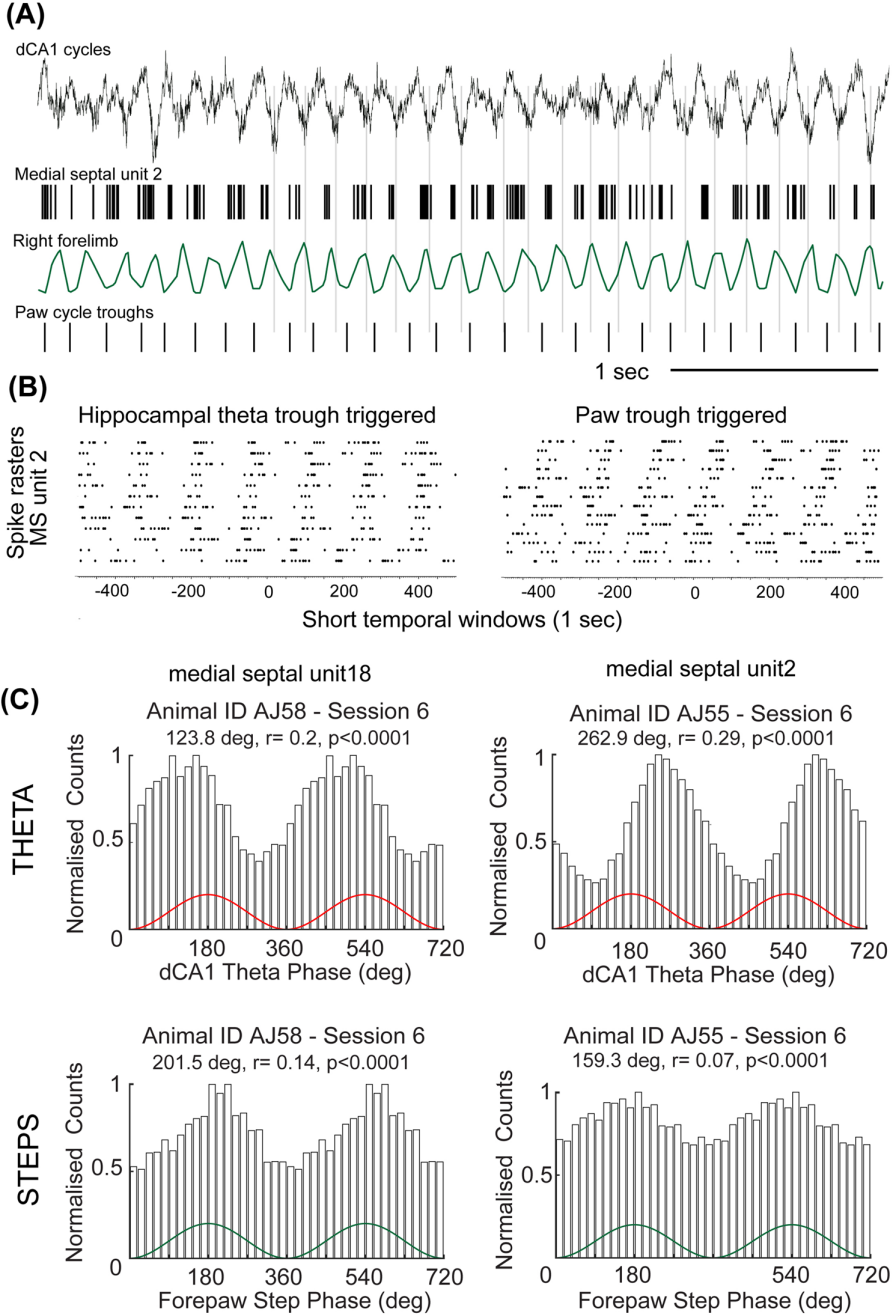
Firing of single medial septal neurons are phase-related to theta and step-cycles. **a** Spike timing of a medial septal theta rhythmic neuron shows simultaneous relationship to both the step-cycles and theta oscillatory cycles. **b** (Left) Hippocampal theta-trough triggered and (right) paw movement cycle trough triggered spike averages of MS unit2 show that the spike trains of a medial septal rhythmic neuron are organized around both the paw and hippocampal theta troughs in short windows. **c** Phase histograms of two medial septal rhythmic neurons simultaneously recorded during theta (top) and stepping (bottom) cycles (data are duplicated to illustrate rhythmicity)

Because the medial septum strongly influences theta rhythmicity in the temporal cortex, we also tested whether the activity of single medial septal neurons was related to the step-cycle. Some of these neurons are highly rhythmic (Dragoi et al. 1999; King et al. 1998). The medial septum integrates and relays rhythmic signals from various brainstem and midbrain nuclei to the temporal cortex (Kaifosh et al. 2013; Kocsis et al. 2001; Kocsis and Vertes 1997; Vertes et al. 2001), and may be one of the routes through which the hippocampo-entorhinal cortical network receives information about self-motion (Campbell and Giocomo 2018; Fattahi et al. 2018; Justus et al. 2017). In the mouse, we identified two types of distinct high-rhythmic firing medial septal neurons based on their activity patterns, molecular parameters and cortical synaptic target preferences (Joshi et al. 2017; Viney et al. 2018). Teevra cells fire short burst duration action potentials maximally at the trough of dorsal hippocampal theta oscillatory cycles and Komal cells fire long bursts of action potentials maximally at the peak of theta oscillatory cycles (Joshi et al. 2017). To analyze their relationship to movement, first we obtained step cycles from a video (AJ27b, sampled at 30 Hz) recorded in the experiments reported earlier (Joshi et al. 2017). We observed a striking coupling between some of the bursts of Teevra and Komal cell firing and the times when the paw touched the ground (Online Video 1). In this recording, the peak running frequency was 4 Hz and we observed that the simultaneously identified rhythmic Teevra (*n* = 5 cells) and Komal cells (*n* = 6 cells) showed burst firing within these 4 Hz cycles. In our current recordings (step cycles obtained using PixyMon), we also observed a similar relationship between rhythmically firing medial septal neurons (*n* = 12 cells) and step cycles. Single medial septal neurons were simultaneously coupled to both the rhythmic theta and paw signals at different running speeds (Fig. 3a). The coupling of medial septal single-cell spiking was maintained for both theta- and step- cycles over grand averages of variable frequencies (Fig. 3b). During the periods when we could reliably capture hippocampal theta activity, step-cycles and rhythmic medial septal cell spiking simultaneously, we did not observe a consistent correlation between the coupling strength of septal cells to theta- and step- cycles (*p* = 0.25, *n* = 12 cells), i.e. neurons with high spike-to-theta coupling did not necessarily have higher spike-to-step coupling. Nevertheless, we note that rhythmic medial septal cells with the highest theta-coupling tended to have lower step-coupling than cells with medium theta-coupling (e.g. in Fig. 3c). Taken together, these results suggest that step-cycle, hippocampal theta and rhythmic firing of medial septal neurons are transiently coupled during short periods distributed throughout movement behavior.

## Discussion

Behavioral engagement of an animal with the environment requires recruitment of many sensory, autonomic, cognitive and musculo-skeletal systems. Analysis of rhythmic oscillatory brain activity shows the temporary coupling and uncoupling of different rhythm generators, e.g. recent studies show the coupling of the respiration-rhythm and theta oscillation in diverse brain regions (Ranade et al. 2013; Tort et al. 2018). In the hippocampus of rodents, a global respiration-related rhythm is occasionally phase-locked to ongoing theta oscillatory activity in the hippocampus during a tactile discrimination task (Grion et al. 2016). Like breathing, running is an example of a sensory-motor process and is part of the physiological repertoire of body movements that accompany behavior. Breathing and locomotion are also coupled oscillators. If and how distinct motor-related physiological variables influence cognitive cortical information processing during behavior remains to be further explored (Kőszeghy et al. 2018; Ledberg and Robbe 2011; Lockmann et al. 2016; Wyble et al. 2004). The afferent and efferent connectivity of the medial septum suggests that it is at an important crossroad between sensory-motor and limbic pathways (Bland and Oddie 2001; Tsanov et al. 2014). Indeed, optogenetically activating medial septal glutamatergic circuitry makes animals run faster and entrains high-frequency theta oscillations (Fuhrmann et al. 2015), but the theta entrainment is likely mediated through local inhibitory connections or descending pathways, as stimulating glutamatergic fibers in the fornix does not entrain theta (Robinson et al. 2016).

We have observed that single medial septal neurons are simultaneously related to theta and step-cycles during navigation in mice. A parsimonious explanation is accidental ‘coincidence’, i.e. two oscillators in the same frequency range may be in phase, lead or lag with respect to the other by chance. Our analysis suggests an alternative explanation. The short temporal windows of theta and step synchrony may reflect epochs in the mnemonic network when information carried from the limbs reports their current position, frequency of alternation and the intentional heading of the animal towards a perceived goal, or the recognition of a location. As we observed through single medial septal theta coordinator neurons, specific neurons are engaged in this process in the theta generator network. We also noted that in our small dataset, while all neurons were simultaneously coupled to both theta and step cycles, there was no correlation between the strengths of step-cycle and theta coupling i.e. neurons with the highest step cycle coupling, on average, did not show the highest theta-coupling. This could indicate that separate populations of medial septal cells specialized to coordinate theta rhythmic population activity and stepping related locomotor activity in the navigation system. This can be interpreted in concert with a recent observation in motor cortical neurons, where strong neuron to population coupling implied weak neuron to body-movement coupling (Kells et al. 2019). From an evolutionary perspective, body movement variables may provide temporal windows for synchronizing neural computations in body-relevant timescales, crucial for behavior. Interestingly, the absence of rhythmic theta activity in flying bats (Yartsev et al. 2011) may be related to the absence of rhythmic limbed locomotion. Furthermore, rhythmicity may not be essential for synchronizing neural activity, but instead an up and down fluctuation around the mean may be relevant for behavior (Kleinfeld et al. 2016). An interaction of the rhythmic stepping locomotor pathway with medial septal theta coordinating and cortical effector networks may be a reason why medial temporal lobe theta oscillatory activity accompanies ambulatory movement in humans (Aghajan et al. 2017). Our simple experiments could not resolve the behavioral state of the animal in finer detail. Further studies in freely behaving animals performing goal-directed tasks are needed to explore the relationship between various movement-related physiological parameters and the activity of single neurons in different areas of the septo-thalamic-temporal cortex cognitive memory network. Such tests can be used to dissect further the nature of neuronal integration in epochs of synchrony.

## Methods

Extracellular electrophysiological recordings were performed in adult male C57Bl6/J mice using multichannel silicon probes (*n* = 4 mice; one mouse also used in Joshi et al. 2017 for different analysis). Mice ranged between 3 and 6 months of age at the time of surgery and were housed with littermates until surgical implantation of the head-plate. All procedures involving experimental animals were under approved personal and project licenses in accordance with the Animals (Scientific Procedures) Act, 1986 (UK) and associated regulations.

### Surgery

Mice were deeply anesthetized with isoflurane (induction chamber 3–4% v/v with airflow and reduced to 1–2% v/v after the animal was positioned in the stereotaxic apparatus) and given a subcutaneous injection of buprenorphine (Vetergesic, 0.08 mg/kg) to expose the skull under aseptic conditions. Three sites were marked: the bregma, MS craniotomy [antero-posterior (AP): + 0.86 mm, medio-lateral (ML): 0 mm, dorso-ventral (DV): 3.5 mm, 0] and hippocampal craniotomy (AP: − 2.4 mm, ML: 1.7 mm, DV: 1.2 mm, 10 postero-anterior angle). The head-plate (1.1 g version, custom made at the Department of Physics, Oxford University) used for head-fixation was secured to the skull using dental cement and three small M1 screws. Two such screws were fixed to the skull above the cerebellum and served as the ground and electrical reference for the recordings.

### Behavioral procedures

Upon recovery from the surgery (typically 3–4 days), mice were trained to run on an air-flow suspended styrofoam ball. Food restriction (to 90% of initial pre-surgery body weight) was aimed to motivate running and mice received small drops of sucrose (20% solution) reward upon reaching the end of the linear maze (jetball, PhenoSys). In addition to the described habituation and training, *n* = 3 mice were also habituated with paw-painting as in Nashaat et al. (2017).

### Recordings and single unit identification

The duration of the recording sessions was determined by the motivational state of the animal as grooming removed the body-paint and impaired paw-tracking, so the sessions typically lasted 20–30 min. For monitoring theta oscillation in the LFP, contacts were aimed at the hippocampal pyramidal cell layer or in stratum oriens in CA1 and referenced to a screw in contact with the dura above the cerebellum; the trough was set as 0. Wide-band (0.1–6000 Hz; 20 kHz sampling rate) recordings were performed using a two-shank acute silicon probe (150 µm intershank distance; two tetrodes per shank; 25 µm spacing between contacts within a tetrode, Neuronexus) connected to a RA16-AC preamplifier (Tucker-Davies). Recordings were then digitally high pass filtered (0.8–5 kHz) and neuronal spikes were detected using a threshold crossing based algorithm. Detected spikes were automatically sorted using the algorithm implemented in KlustaKwik (Kadir et al. 2014), followed by manual adjustment of the clusters (Csicsvari et al. 1999) to obtain well-isolated single units, based on cross-correlations, spike-waveform and refractory periods. Multiunit or noise clusters or those with less than 300 spikes were discarded from analysis.

### Electrophysiological data analysis

Categories of running episodes: Periods in which both theta and step signal were recorded were included in the analysis. RUN periods were parsed in accordance with the following conditions (1–3), excluding parsed data with < 200 step-cycle troughs.

1. *Running conditions* (a) when the virtual reality feedback was OFF (VRoff), typically at the beginning and end of the recording sessions; (b) when the virtual reality feedback was ON (VRon), typically in the middle of the recording sessions; and (c) run times during the approach and consumption of reward (VRrwd), arbitrarily defined as 1 s before and after reward delivery.
2. *Running speed dependent parcellation* RUN periods were segmented into three speed bins: slow (2–7 cm/s), medium (7–13 cm/s) and fast (13–19 cm/s), which represented 95% of the distribution of running speeds in our experiments.
3. All running time was divided into 500 ms bins and the number of theta troughs and step-cycle troughs were counted for each bin. Then, all the bins were assigned a tag 'steps troughs > theta troughs', 'step troughs = theta troughs' or 'step troughs > theta troughs'.

For each recorded neuronal spike and step-cycle trough, we calculated the mean depth of modulation and the preferential mean phase of occurrence using Rayleigh’s method (Zar 1999). Briefly, a theta phase was attributed to each step-cycle-trough and the resulting distribution was regarded as theta modulated if Rayleigh test indicated that the phases were not distributed uniformly around the theta cycles (*p* < 0.05). For each condition, we quantified the depth of modulation by summing all phases as unity vectors; the resulting vector sum was normalized by the number of step-cycle troughs (Zar 1999) using MATLAB circular statistics toolbox. The length of the normalized vector (“*r*”) can range from 0 (uniform distribution) to 1 (all phase angles identical). The direction of *r* indicates the step-cycle-trough times' mean phase angle. Phase histograms (18° bins) represent step-cycle trough timing relative to the theta oscillation troughs.

*Shuffling analysis* To assess the statistical significance of our step-theta coupling, we used a shuffling analysis as follows. We offset the stepping data (i.e. the continuous signal depicting paw displacement) randomly by 1–3 s, calculated the step-cycle troughs and determined the mean vector lengths with respect to theta as before. This procedure was repeated 500 times to obtain distributions of shuffled mean vector lengths and corresponding *p* values. This distribution was compared to the original dataset. Values of obtained mean vector length in the original dataset outside 99% confidence interval of the shuffled distribution were considered significant.

### Paw tracking

In three animals, the forepaws of mice were painted with two different colors, one for each paw (green and red). A Pixy camera (Charmed labs, Carnegie Melon University) was equipped with a 10–30 mm f1.6 IR lens and connected to the USB port of a computer as described in Nashaat et al 2017. Pixy uses an HSV (hue, saturation and value) color-based filtering algorithm to track colored objects. Color signatures were tuned to achieve consistent tracking during each experiment. The color signature was mapped via a serial port to Spike2 using a freely available software developed by Nashaat et al (2017) called signature mapper. In addition, another camera (Supereyes) was used to record the video of the animal from a different angle at 30 frames per second. Step cycles were obtained by tracking the right paw of the animal (painted green) using ‘tracking’ plugin in Fiji (Schindelin et al. 2012). Step cycles obtained using the two methods were highly correlated. The current study has limitations, as reported in the Nashaat et al. (2017). It has a lag of ~ 30 ms and may be the reason of some of the variability observed. Using manual tracking of the paws of the animal, we reproduced our observations of the coupling during short temporal segments, though this method was limited by even lower sampling rate (30 Hz). As a complimentary approach, we used a recently published machine learning algorithm, DeepLabCut (Mathis et al. 2018) to extract the paw position from recorded videos sampled at 30 frames per second. 100 frames were selected using '*k*-means' clustering for labeling to represent the diversity of stepping observed on the jetball. The network was trained for > 200,000 iterations until the loss reached a plateau. Segments of recording with likelihood estimate > 0.9 were used for analyzing the power spectral features of the stepping cycle. Note, that this method can only be used to study the range of frequencies of stepping in head-fixed mice in our experimental setup. This is a consequence to the timing of each camera frame, which cannot be attributed to a precise time in the recording file due to undocumented lost frames during acquisition.

## Electronic supplementary material

Below is the link to the electronic supplementary material.

Supplementary file1 (MP4 15551 kb)

## References

[CR1] Aghajan M, Schuette Z, Fields P, Tran TA, Siddiqui ME, Hasulak SM, Suthana NR (2017). Theta oscillations in the human medial temporal lobe during real-world ambulatory movement. Curr Biol.

[CR2] Alcantara S, Ruiz M, D'Arcangelo G, Ezan F, de Lecea L, Curran T, Sotelo C, Soriano E (1998). Regional and cellular patterns of reelin mRNA expression in the forebrain of the developing and adult mouse. J Neurosci.

[CR3] Bland BH, Oddie SD (2001). Theta band oscillation and synchrony in the hippocampal formation and associated structures: the case for its role in sensorimotor integration. Behav Brain Res.

[CR4] Campbell MG, Giocomo LM (2018). Self-motion processing in visual and entorhinal cortices: inputs, integration, and implications for position coding. J Neurophysiol.

[CR5] Csicsvari J, Hirase H, Czurko A, Mamiya A, Buzsaki G (1999). Oscillatory coupling of hippocampal pyramidal cells and interneurons in the behaving rat. J Neurosci.

[CR6] Czurkó A, Huxter J, Li Y, Hangya B, Muller RU (2011). Theta phase classification of interneurons in the hippocampal formation of freely moving rats. J Neurosci.

[CR7] Dahl CJ, Lutz A, Davidson RJ (2015). Reconstructing and deconstructing the self: cognitive mechanisms in meditation practice. Trends Cogn Sci.

[CR8] Dragoi G, Carpi D, Recce M, Csicsvari J, Buzsaki G (1999). Interactions between hippocampus and medial septum during sharp waves and theta oscillation in the behaving rat. J Neurosci.

[CR9] Fattahi M, Sharif F, Geiller T, Royer S (2018). Differential representation of landmark and self-motion information along the CA1 radial axis: self-motion generated place fields shift toward landmarks during septal inactivation. J Neurosci.

[CR10] Fuhrmann F, Justus D, Sosulina L, Kaneko H, Beutel T, Friedrichs D, Schoch S, Schwarz MK, Fuhrmann M, Remy S (2015). Locomotion, theta oscillations, and the speed-correlated firing of hippocampal neurons are controlled by a medial septal glutamatergic circuit. Neuron.

[CR11] Gothe NP, Hayes JM, Temali C, Damoiseaux JS (2018). Differences in brain structure and function among yoga practitioners and controls. Front Integr Neurosci.

[CR12] Grillner S, Wallen P (1985). Central pattern generators for locomotion, with special reference to vertebrates. Annu Rev Neurosci.

[CR13] Grion N, Akrami A, Zuo Y, Stella F, Diamond ME (2016). Coherence between rat sensorimotor system and hippocampus is enhanced during tactile discrimination. PLoS Biol.

[CR14] Jacobs J, Kahana MJ, Ekstrom AD, Fried I (2007). Brain oscillations control timing of single-neuron activity in humans. J Neurosci.

[CR15] Jeewajee A, Barry C, O'Keefe J, Burgess N (2008). Grid cells and theta as oscillatory interference: electrophysiological data from freely moving rats. Hippocampus.

[CR16] Joshi A, Salib M, Viney TJ, Dupret D, Somogyi P (2017). Behavior-dependent activity and synaptic organization of septo-hippocampal GABAergic neurons selectively targeting the hippocampal CA3 area. Neuron.

[CR17] Justus D, Dalugge D, Bothe S, Fuhrmann F, Hannes C, Kaneko H, Friedrichs D, Sosulina L, Schwarz I, Elliott DA, Schoch S, Bradke F, Schwarz MK, Remy S (2017). Glutamatergic synaptic integration of locomotion speed via septoentorhinal projections. Nat Neurosci.

[CR18] Kadir SN, Goodman DFM, Harris KD (2014). High-dimensional cluster analysis with the masked em algorithm. Neural Comput.

[CR19] Kaifosh P, Lovett-Barron M, Turi GF, Reardon TR, Losonczy A (2013). Septo-hippocampal GABAergic signaling across multiple modalities in awake mice. Nat Neurosci.

[CR20] Kells PA, Gautam SH, Fakhraei L, Li J, Shew WL (2019). Strong neuron-to-body coupling implies weak neuron-to-neuron coupling in motor cortex. Nat Commun.

[CR21] King C, Recce M, O'Keefe J (1998). The rhythmicity of cells of the medial septum/diagonal band of Broca in the awake freely moving rat: relationships with behaviour and hippocampal theta. Eur J Neurosci.

[CR22] Kleinfeld D, Deschênes M, Ulanovsky N (2016). Whisking, sniffing, and the hippocampal θ-rhythm: a tale of two oscillators. PLoS Biol.

[CR23] Kocsis B, Vertes RP (1997). Phase relations of rhythmic neuronal firing in the supramammillary nucleus and mammillary body to the hippocampal theta activity in urethane anesthetized rats. Hippocampus.

[CR24] Kocsis B, Di Prisco GV, Vertes RP (2001). Theta synchronization in the limbic system: the role of Gudden's tegmental nuclei. Eur J Neurosci.

[CR25] Kőszeghy Á, Lasztóczi B, Forro T, Klausberger T (2018). Spike-timing of orbitofrontal neurons is synchronized with breathing. Front Cell Neurosci.

[CR26] Kropff E, Carmichael JE, Moser MB, Moser EI (2015). Speed cells in the medial entorhinal cortex. Nature.

[CR27] Ledberg A, Robbe D (2011). Locomotion-related oscillatory body movements at 6–12 Hz modulate the hippocampal theta rhythm. PLoS ONE.

[CR28] Lockmann ALL, Laplagne DA, Leão RN, Tort AB (2016). A respiration-coupled rhythm in the rat hippocampus independent of theta and slow oscillations. J Neurosci.

[CR29] Mathis A, Mamidanna P, Cury KM, Abe T, Murthy VN, Mathis M, Bethge M (2018). DeepLabCut: markerless pose estimation of user-defined body parts with deep learning. Nat Neurosci.

[CR30] McNaughton BL, O'Keefe J, Barnes CA (1983). The stereotrode: a new technique for simultaneous isolation of several single units in the central nervous system from multiple unit records. J Neurosci Methods.

[CR31] Mizuseki K, Sirota A, Pastalkova E, Buzsaki G (2009). Theta oscillations provide temporal windows for local circuit computation in the entorhinal-hippocampal loop. Neuron.

[CR32] Musall S, Kaufman MT, Juavinett AL, Gluf S, Churchland AK (2019). Single-trial neural dynamics are dominated by richly varied movements. Nat Neurosci.

[CR33] Nashaat MA, Oraby H, Blanco L, Dominiak S, Larkum ME, Sachdev RNS (2017). Pixying behavior: a versatile real-time and post-hoc automated optical tracking method for freely moving and head fixed animals. eNeuro.

[CR34] Nerad L, McNaughton N (2006). The septal EEG suggests a distributed organization of the pacemaker of hippocampal theta in the rat. Eur J Neurosci.

[CR35] Phillips-Silver J, Trainor LJ (2005). Feeling the beat: movement influences infant rhythm perception. Science.

[CR36] Ranade S, Hangya B, Kepecs A (2013). Multiple modes of phase locking between sniffing and whisking during active exploration. J Neurosci.

[CR37] Robinson J, Manseau F, Ducharme G, Amilhon B, Vigneault E, El Mestikawy S, Williams S (2016). Optogenetic activation of septal glutamatergic neurons drive hippocampal theta rhythms. J Neurosci.

[CR38] Schindelin J, Arganda-Carreras I, Frise E, Kaynig V, Longair M, Pietzsch T, Preibisch S, Rueden C, Saalfeld S, Schmid B (2012). Fiji: an open-source platform for biological-image analysis. Nat Methods.

[CR39] Tort ABL, Ponsel S, Jessberger J, Yanovsky Y, Brankack J, Draguhn A (2018). Parallel detection of theta and respiration-coupled oscillations throughout the mouse brain. Sci Rep.

[CR40] Tsanov M, Chah E, Reilly R, O'Mara SM (2014). Respiratory cycle entrainment of septal neurons mediates the fast coupling of sniffing rate and hippocampal theta rhythm. Eur J Neurosci.

[CR41] Vertes RP, Albo Z, Di Prisco GV (2001). Theta-rhythmically firing neurons in the anterior thalamus: implications for mnemonic functions of papez's circuit. Neuroscience.

[CR42] Viney TJ, Salib M, Joshi A, Unal G, Berry N, Somogyi P (2018). Shared rhythmic subcortical GABAergic input to the entorhinal cortex and presubiculum. eLife.

[CR43] Wyble BP, Hyman JM, Rossi CA, Hasselmo ME (2004). Analysis of theta power in hippocampal EEG during bar pressing and running behavior in rats during distinct behavioral contexts. Hippocampus.

[CR44] Yartsev MM, Witter MP, Ulanovsky N (2011). Grid cells without theta oscillations in the entorhinal cortex of bats. Nature.

[CR45] Zar JH (1999). Biostatistical analysis.

